# Factors influencing the migration of West African health professionals

**DOI:** 10.11604/pamj.2016.24.237.9402

**Published:** 2016-07-14

**Authors:** Mat Lowe, Duan-Rung Chen

**Affiliations:** 1 Institute of Health Policy and Management, College of Public Health, National Taiwan University, Taiwan (R.O.C)

**Keywords:** Migration, West Africa, West African Health Organization, young professionals

## Abstract

**Introduction:**

The West African health sector is characterized by a human resource base lacking in numbers and specialized skills. Among the contributory factors to this lack of human resource for health workforce include but not limited to the migration of health professionals.

**Methods:**

This cross-sectional survey targeted 118 young professionals who have participated in the Young Professional Internship Program (YPIP) of the West African Health Organization (WAHO), from (2005-2013). It inquired about their socio-demographic characteristics associated with migration and reasons for going to their preferred or most likely destinations through online survey.

**Results:**

Of the 118 young professionals, 100 responded to the online survey, of which (28%) have migrated and (72%) did not migrate. Migration was more common among males and those (age≤31 years old), single with high dependency level and no previous work experience. Having a medical profession and being posted to urban or semi-urban area was also associated with their emigration. Their most important reasons for going to preferred or most likely destinations were to have fair level of workload, job promotion and limited occupational risks.

**Conclusion:**

This finding suggests that the migration of health professionals is situation dependent, mediated by basic socio-demographic variables and work related conditions. These issues have implications for curbing the brain drain potential of health professionals in the West African health sector.

## Introduction

The West African health sector is characterized by a human resource base lacking in numbers and specialized skills [1]. A major contributory factor to this lack of human resource for health workforce includes but not limited to the migration of health professionals to rich countries. Anecdotal evidence suggests that West African-trained physicians in particular have been migrating primarily to the US and the UK since the time medical education began in Nigeria and Ghana in the 1960s [2]. Since then, a flurry of scholarly work has been devoted to understanding the migration patterns of health professionals from sub-Saharan African countries. Many studies have revealed a number of factors responsible for the migration of health professionals. These include career development-related factors [3], financial considerations such as better salary [4], poor and difficult working conditions, including long working hours [5], high patient workload and inadequate resources [6]. Political instability [7] and other personal related factors such as perceived social status and lifestyle [8], as well as the prevalence of diseases such as HIV/AIDS [3] have also been documented. Although many of these studies have accurately identified the factors influencing the migration of health professionals; they have been largely anecdotal and provide little information about the mediating role of demographic variables such as age, gender, profession, marital status and household dependency level. Labor mobility and social demographic studies have shown that individual-level factors such as gender, age, work experience, marital status, social groups and relationship including migration network and families are important correlates in the decision to migrate [9–12]. However, very little of this information has been explored within the context of West African health professionals. This study is based on the Young Professional Internship Program (YPIP) of the West African Health Organization (WAHO). The YPIP is a training program of the Division of Human Resource Development of WAHO. It was developed in 2005 by WAHO and its partners in recognition of the lack of adequate human resource for health in West Africa, with the primary objective of providing young professionals with knowledge, practical skills and experience for sustainable management of health issues in West Africa [1]. It includes basic training in management and principles of public health, language (English and French), and information communication technology. In addition, interns are also sent out to different West African countries for practical work experience on reproductive health and HIV/AIDS program management, malaria, child survival, prevention of blindness, health service research and disease control. Since its inception in 2005, the program has graduated 118 young professionals across fifteen member countries of the Economic Community of West African States (ECOWAS). Evaluation research suggested that YPIP has substantial potential to contribute to strengthened health systems [1]. However, no empirical study has been conducted to examine the emigration potential of young professionals who have graduated from the program. This study aims to examine whether socio-demographic characteristics such as sex, age, marital status, dependency level, professional occupation, work experience and region will make important difference in the migration of health professionals who have participated to the program. The study also investigates some of the most important reasons for going to their preferred or most likely destinations.

## Methods

**Design:** This study is a cross-sectional survey of West African health professionals who have participated to Young Professional Internship Program of the West African Health Organization (WAHO). It employed the use of online survey questionnaire as data collection method.

**Figure 1:**
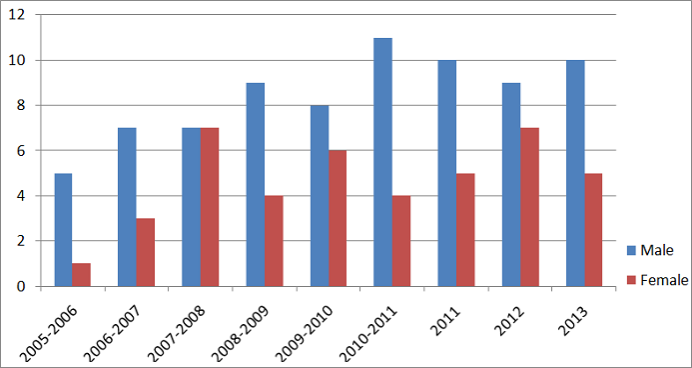
Distribution of interns by sex and year

**Study participants:** Our target population consisted of all young professionals who have participated in the YPIP from 2005-2013. At the time of the survey, there were 118 young professionals who have participated to the program. The program as a rule, accepts 15 interns each year; 1 from each of the 15 ECOWAS countries. However, where there are no qualified applicants from one or more countries, more than one may be selected from a single country. The number of interns in the program has grown since the start of the program in 2005 (Figure 1).

**Data collection:** All the 118 young professionals on the list of the YPIP alumni page were invited by email with a link to complete a web-based online survey questionnaire. Others who were not member of the alumni page were contacted by email with attached link to the survey. The program alumni database, spreadsheet of contact information and handbook from WAHO were also searched for additional information. The questionnaires for the data collection were pre-tested on three YPIP participants prior to administration. Following this pre-testing, the questionnaires were then put online on YPIP alumni page. All the questionnaires were close ended written entirely in English since all the respondents were fluent in both spoken and written English, although their countries speak different official languages (such as English, French and Portuguese). The questionnaires took typically 6-10 minutes to complete and asked questions relating to: (1) Respondents' socio-demographic characteristics (such as sex, age, dependency level, professional occupation, work experience and posting region). This set of basic socio-demographic variables was evaluated against the two main dependent variables of interest (migration or no migration) to see if there were important differences. The questionnaires also included additional questions about important reasons for going to preferred or most likely destinations. The responses to this question were ranked against five-point Likert scale from very important to not important at all, no comment, less important and least important), and included reasons such as: to have better salary or income, risk and other allowances, health insurance coverage, control over professional practice, suitable job match, opportunities for career development, higher life satisfaction, fair level of workload, self-actualization, better health condition, social security and other retirement benefits such as family support; adequate equipment/or materials to work with, improved working conditions, limited occupational risks and fair level of job promotion.

**Variable studied:** The dependent variables for the study included migration or no migration. Migration is defined as when a respondent’s country of first job position is different from his or her second job position following graduation from YPIP. We considered second job country to include not only countries outside of West Africa (international), but also within (intra) Africa since YPIP was designed specifically to address the lack of human resource for health workforce in the West Africa region. No migration is defined as when a respondent’s first job position is the same as his or her second job position following graduation from YPIP. This means migration did not take place or only internally within the same country. Respondents were asked to provide two job positions they have held and for each job to specify which country. First job position means a job title or position a respondent has held prior to participating in the YPIP. Second job position was considered as job title or position a respondent has occupied after he or she has graduated from YPIP. The different countries provided by respondents were coded (in numbers) and if the code for the country of first job position differs from the second job position, it means the respondent has changed job at country of first job position to second job position in another country. Our independent variables included are sex, age, dependency level, professional occupation, work experience and posting region. The variable for sex was coded as 1 for male and 0 for female. The age category was divided into (≤31; 32-34; and ≥35 respectively). Dependency level was classified into two different categories: low and high dependence. High dependency means a respondent has many people (such as a wife, husband, father or mother) to support and the opposite is true for low dependency. Professional occupation was divided into two categories: medical and public health officer. A medical officer was considered to be a medical doctor whereas the public health officer category comprises of public and environmental health officers and other allied health professionals such as nurses, laboratory technicians, pharmacists, nutritionist and biologists. This combination was done because there were few observations for allied health professionals. The data distinguish if a respondent was employed with a “yes” response, unemployed with “no” response, studying or in other type of economic inactivity as “others”. The variable of work experience was obtained from this construction, in which, respondents were asked if they have had previous work experience in the public or private health sector prior to participating in the YPIP or after graduation from the program. Posting region was divided into three categories: rural (coded as 1), urban (2) and semi-urban areas as (3). However, in the analysis we combined urban and semi-urban together because there were few observations for semi-urban.

**Statistical analysis:** We first conducted a descriptive analysis of respondents' socio-demographic characteristics and of the outcome variables of interest (migration or no migration) using mean and standard deviation for continuous variables and frequencies and percentages for categorical variables [13]. All the variables that correlate with migration were entered. Second, we conducted bi-variable analyses using the Chi-Square test to determine which socio-demographic variables were associated with each of the outcome variables of interest (migration or no migration). The adjusted OR was estimated by entering all the variables simultaneously, whereas the crude OR was estimated by entering all variables one-by-one. The 95% confidence intervals were calculated using fisher's exact test P for variables less than or equals to five. We considered two-sided p-values and no statistical significant differences at the P=0.05 level. For the additional question about important reasons for going to preferred or most likely destinations, the number of responses to each category equals (100). Five responses were provided. The responses were summarized into scores, mean and standard deviation. Higher variability in the standard deviation means different responses and when the variability is low, it means respondents gave similar responses or responded close to the mean. Also, if the variability is not very high, it means most of the respondents responded close to very important. Microsoft Office Excel 2007 was used for data management and IBM SPSS Statistics 20 for data analyses. The study was approved by the West African Health Organization (WAHO) YPIP management team. Informed consent was also obtained for individual participation from study respondents.

## Results

Of the 100 young professionals who responded to the online survey, (28%) have changed job position from their country of first job to second job position in another country following graduation from YPIP. This means they have graduated. The remaining (72%) did not change their first job position in another following graduation from YPIP, which means they did not migrate or have remained in the same country for their first job and second job positions. However, the fact that close to (30%) of the young professionals has changed their first job position to second job position in another country following graduation from YPIP shows that there is high emigration potential among them. Of the 100 young professionals who responded to the survey the majority (72%) were males and (28%) were females. Their age ranges from ≤31 to ≥35 years old. About (18%) were single and (82%) were married. The majority (70%) have high dependency level from their family members, while the remaining (30%) have low dependency levels. The largest category by profession was public health officers (74%), followed by medical doctors (26%). Of the 100 young professionals (82%) have previous work experience in the public or private health sector prior to participating in the YPIP and only (18%) of them had no previous work experience in the public or private health sector prior to participating in the YPIP. Of the 100 young professionals (78%) were posted to urban or semi-urban areas, while (22%) were posted to rural areas (Table 1). This suggests that these health professionals are distributed in a way which disadvantaged rural population in their countries. In the bi-variable analyses, we evaluated the basic set of demographic variables (sex, age, dependency level, professional occupation, work experience and region) against the two main dependent variables (migration or no migration) to see if there were important differences. This evaluation revealed no statistical significant differences at the P=0.05 level, which could be due to the effect of low statistical power considering the sample size of the study. However, this was not true in the logistic regression results (Table 2). Although not very strong, we found slight differences. It was that migration was more common among males than females. With regard to age, compare with the reference category (i.e. age ≤31), migration was less common among young professionals age (≥35) years old. This effect could be because relatively older young professionals are close to their retirement age, but it could also be due to the grouping of the respondents. We also found that compared with being single, migration was less common among married respondents (OR=0.910; 95% CI 0.172-4.825). Also, compare to those with low level of dependency, those with high level of dependency were more migratory (OR=1.99; 95% CI 0.561-7.096). We also found that compare with public health officers, migration was more common among medical doctors (OR=1.48; 95% CI 0.42-5.170). Being posted to urban or semi-urban area was statistically associated with migration (OR=3.676; 95% CI 1.118-12.08). In terms of attitude to migration (Table 3), we asked respondents to respond on a five-point Likert scale on some of the most important reasons for going to their preferred or mostly likely destinations. We found some variability in responses, which were sorted out starting first with the highest or most attractive responses from the respondents. The most attractive response with the highest score was to have fair level of workload with a mean score of (2.48), followed by to have fair level of job promotion and limited occupational risks respectively. The least ranked response or bottom score was to have career prospects with a mean score of (1.41). This result could be because these young professionals have already obtained the needed professional skills for their work from their previous training and the YPIP, which could facilitate their opportunities for career prospects or entry into the labor market compared with other health professionals who have not participated to such programs as the YPIP.

**Table 1:** Respondents’ profile (%)

**Sex**	
Male	72 (72.0)
Female	28 (28.0)
**Age (years)**	
≤31	25 (25.0)
32-34	42 (42.0)
≥35	33 (33.0)
**Marital status**	
Single	18 (18.0)
Married	82 (82.0)
**Dependency level^+^**	
High	70 (70.0)
Low	30(30.0)
**Professional Occupation**	
Public health officer^+^	74 (74.0)
Medical officer	26 (26.0)
**Work experience**	
Yes	82(82.0)
No	18 (18.0)
**Posting region**	
Rural/semi urban	22 (22.0)
Urban	78 (78.0)

Whether a person has many (high) or (low) less people (such as a wife, husband, father or mother) he or she is supporting

+ Public health officer category comprised of public and environmental health and other allied health professionals.

**Table 2:** Logistic regression results

Variables	Crude OR^a^ 95 % CI^†^		Adjusted OR^b^ 95% CI^†^	
**Sex**				
Female^₸^	1		1	
Male	1.83	0.54 - 6.12	2.02	0.564-7.257
**Age**				
≤31^₸^	1		1	
32-34	0.75	0.18-3.01	0.385	0.076-1.957
≥35	2.12	0.56 - 8.01	0.277	0.083-0.917
**Marital status**				
Single^₸^	1		1	
Married	1.17	0.29 -4.64	0.910	0.172-4.825
**Dependency level**				
Low^₸^	1		1	
High	2.0	0.60 -6.67	1.99	0.561-7.096
**Professional occupation**				
Public health officer^₸^	1		1	
Medical doctor	2.0	0.18 -22.05	1.48	0.42-5.170
**Work experience**				
Yes	1		1	
No	4.25	0.52 -34.74	1.069	0.339-3.373
**Posting region**				
Rural^₸^	1		1	
Urban/ Semi-urban	1.63	0.53 - 5.01	3.676	1.118-12.08

a Crude odds ratio were estimated by entering all variables one-by-one

b Adjusted odds ratio was estimated by entering all variables simultaneously

† The 95% confidence intervals were calculated using fisher’s exact test P.

₸ Reference group

**Abbreviation:** OR, odds ratio; CI, confidence intervals

**Table 3:** Mean reasons for going to preferred or most likely destination

Variables	Mean	SD
To have fair level of work load	2.48	1.32
To have fair level of job promotion	2.43	1.402
To have limited occupational risks	2.43	1.217
To have health insurance coverage	2.21	1.31
To get promoted when due for promotion	2.14	1.15
To have family or social support	2.13	1.212
To have social security	2.05	1.132
To have control over my professional practice	2.04	1.21
To have better work and risk allowances	1.87	1.05
To have adequate equipment/or materials to work with	1.81	1.042
To have better life satisfaction	1.81	1.04
To have better health condition	1.80	0.953
To have more academic opportunities	1.69	0.94
To achieve self actualization	1.65	0.84
To find a suitable job match	1.55	0.82
To have better working conditions	1.54	0.834
To have better income or salary	1.53	0.822
To have career prospects	1.41	0.84

**Note:** the number of responses to each category all equals to (100). Higher variability in the standard deviation means different responses and when the variability is low, it means respondents gave similar responses or responded close to the mean.

**Abbreviation:** SD; standard deviation

## Discussion

**Factors associated with the migration of health professionals:** Although there is growing concern in the number of female lead migrants from African countries [14], we found that migration in this study was more common with males than with females. This difference in migration pattern may be due to the restricted geographic mobility of women [15] and their informal care giver responsibility [16]. Research has also shown that many female African migrant health workers are unable to work according to their qualifications due to gender discriminatory policies and the lack of recognition for their qualifications in the country of destination [17]. Our finding postulates that compared with being married, migration was also more common with young professionals who were single. This finding is in consistent with another study conducted in Lebanon [13] but contradicts other studies, such as [18], who argues that the likelihood of migration of unmarried men is higher than it is for single people. We also found that migration was more common among younger respondents age (≤31) years old compared with relatively older respondents, age (≥35) years old. Although we do not have concrete evidence, we believe that relatively older respondents tend to migrate less, not only because the costs of migrating are higher at older ages, but also because the gain in terms of the expected earnings is smaller [19]. Research has also shown that household membership size, relative wealth, and the age and sex of its inhabitants may play a part in the decision to migrate [20]. This confirms our finding that among the respondents; those with high dependency level from their family members were more migratory compared with those that have less family members to support. Therefore, it seems that the possibility of instituting mean-tested family welfare benefits and other allowances may be at the root of reducing the migration of health professionals who have high dependency levels from their family members. The literature has shown that a mix of both financial and non-financial incentives is effective in addressing motivation [21]. This could also have implications for health worker migration. For migration between particular places, [22] argues that the relationship between occupation and migration may not be linear because the observed difference may be a consequence of migration, rather than a constraint to it. We, however, found that professional occupation makes a slightly important difference in the migration pattern of health professionals. Our findings showed that migration was more common among medical doctors compared to public health officers. One plausible explanation for this observed occupational difference in migration pattern could be due to the high demand of medical doctors in developed countries. With regard to work experience, we found that migration was more common among respondents without previous work experience prior to joining the YPIP compared to those with previous work experience. This, in a way, contradicts the notion that more experienced workers are geographically more mobile. We postulates that respondents without previous work experience may be relatively younger and are likely to be single, which unlike married and older respondents may increase their physics cost of moving and decreases the length of the accrual period for benefits, thus inhibiting their geographic mobility [23]. The geographical push factors inhibiting posting preference is also associated with migration. We found that compared with rural posting, being posted to urban or semi-urban area was statistically associated with migration. One plausible explanation for this statistical association could be because migration information is more accessible in urban areas than in rural areas due in part to the consequence of rural-urban inequality in development [24]. All these issues put together might explain at least partly some of the factors influencing the migration of West African health professionals. But other reasons they are drawn abroad have also existed.

**Most important reasons for going to preferred or most likely destinations:** The most important reasons respondents pointed out in their decision to migrate to their preferred or most likely destinations include desire to have fair level of workload, job promotion and limited occupational risks. Similar issues have also been documented in other studies [6, 25], and these have implications for 'taming the brain drain' potential of health professionals in the West African health sector.

**Study limitations:** While the data presented in the paper has provided understanding of some of the factors associated with the migration of health professionals, including their most important reasons for going to their preferred or most likely destinations. It is noteworthy that the study is also limited by its research design, which must be considered when interpreting the findings. First, it is a cross-sectional study design and only reflected the views of health professionals from the Young Professionals Internship Program (YPIP) of the West African Health Organization (WAHO). Therefore, it cannot show vast differentials and could not be generalized beyond this population of West African health professionals. Finally, although the respondents comprised of different health professional categories, other health professionals not part of the YPIP were not included. The study, therefore, recommends a more sophisticated study methodology with a larger sample size.

## Conclusion

Among the most salient points of this study is its multi-national nature and reasonable inclusion of different health professional categories across the fifteen member countries of the Economic Community of West African States (ECOWAS). The study has also provided both post and pre-migration information of health professionals. Pre-migration data, in particular provides valuable information about motivations, perceptions and expectation concerning migration, which could be used by home and recipient countries in the formulation of migration policies [26].

### What is known about this topic

- The West African health sector is characterized by a lack of human resource for health;
- Among the contributory factors to this shortage of human resource for health workforce includes the migration of health professionals to rich countries.

### What this study adds

- That the migration of health professionals is situation dependent, mediated by basic socio-demographic variables and work related conditions;
- These issues have implications for curbing the brain drain potential of health professionals in the West African health sector.
